# Severe Hypophosphatemia Leading to Acute Worsening of Heart Failure and Myopathy: A Case Report

**DOI:** 10.7759/cureus.80769

**Published:** 2025-03-18

**Authors:** Yukifumi Ishikawa, Taku Harada, Kazushi Yamasato, Mori Nakai

**Affiliations:** 1 General Medicine, Nerima Hikarigaoka Hospital, Tokyo, JPN; 2 Diagnostic and Generalist Medicine, Dokkyo Medical University Hospital, Mibu, JPN

**Keywords:** heart failure, myopathy, phosphate levels, severe hypophosphatemia, vitamin d deficiency

## Abstract

This case highlights a rare instance of severe hypophosphatemia precipitating acute exacerbation of heart failure and myopathy in an 88-year-old male patient residing in a long-term care facility. The patient presented with edema and limb weakness, with a background of chronic heart failure, kidney disease, and vitamin D deficiency. Despite initial treatments targeting anemia and heart failure, the patient's condition did not improve until severe hypophosphatemia was identified and treated with phosphate supplementation, leading to rapid clinical improvement. This case highlights the importance of considering hypophosphatemia in the differential diagnosis of worsening heart failure or myopathy, particularly in frail elderly individuals residing in facilities with a high risk of vitamin D deficiency. It suggests that measuring serum phosphate levels should be considered in cases of heart failure or myopathy in such populations to prevent delays in diagnosis and treatment.

## Introduction

Phosphates are essential for various bodily functions such as energy production, bone formation, and cellular signal transduction. Hypophosphatemia, a condition characterized by low phosphate levels in the blood, is defined as mild (2.0-2.5 mg/dL), moderate (1.0-2.0 mg/dL), and severe (<1.0 mg/dL) [1]. Epidemiological studies in hospitalized populations have shown that the prevalence of moderate hypophosphatemia ranges from 2.2% to 3.1%, and severe hypophosphatemia ranges from 0.2% to 0.4% [1]. Additionally, the presence and progression of frailty in older adults are risk factors for hypophosphatemia, with reports indicating that approximately 11% of severely frail patients suffer from hypophosphatemia [2].

The symptoms and signs of hypophosphatemia usually do not appear when the condition is solely due to a shift of phosphate into cells but become apparent in cases of chronic severe intracellular phosphate depletion, with levels <1 mg/dL [3]. Symptoms can vary widely, including neuromuscular symptoms (weakness, sensory disturbances, rhabdomyolysis, encephalopathy, seizures), left ventricular dysfunction, respiratory failure, and hemolysis, and are primarily observed in situations such as chronic alcohol use disorder, refeeding syndrome, and diabetic ketoacidosis [3]. Severe hypophosphatemia can cause proximal muscle-dominant myopathy and cardiac dysfunction [4-7]. However, serum phosphate levels are rarely measured routinely as part of the diagnostic workup for myopathy or worsening heart failure.

In this case, we encountered a patient who developed worsening heart failure and myopathy due to severe hypophosphatemia, believed to be caused by vitamin D deficiency due to institutionalization. As there are no specific symptoms or findings for hypophosphatemia, measuring serum phosphate levels could be clinically significant as part of the diagnostic workup for worsening heart failure and proximal muscle-dominant myopathy in older adults.

## Case presentation

**Figure 1:**
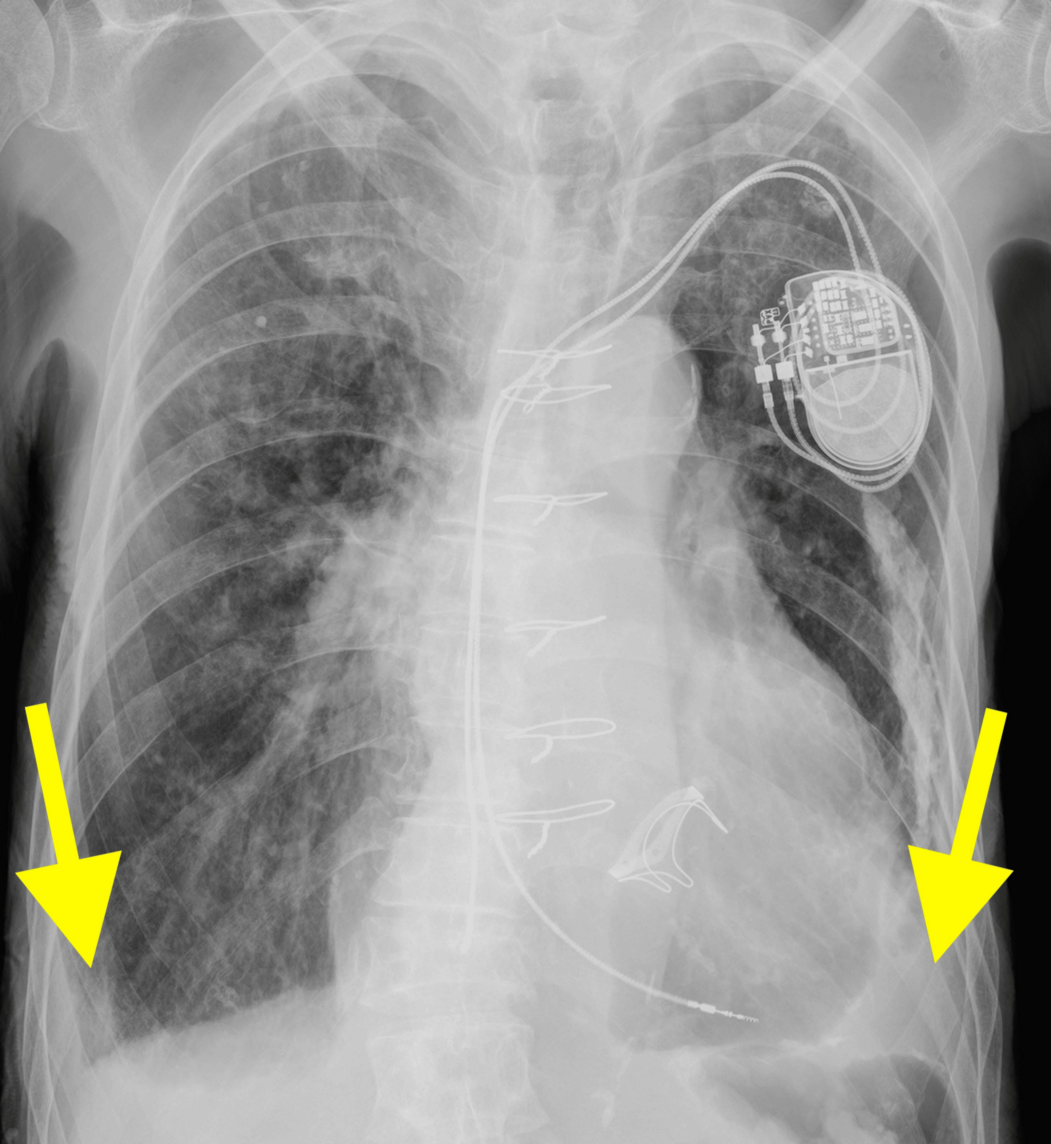
Chest X-ray image upon admission The chest X-ray reveals cardiomegaly and blunting of the costophrenic angles due to bilateral pleural effusion (indicated by yellow arrows), along with a less distinct left hemidiaphragm. Additionally, the presence of a pacemaker and post-surgical changes related to heart valve disease are observed.

An 88-year-old male patient residing in a long-term care facility presented to our department with facial edema and limb weakness, which had developed over several weeks. He had been using a wheelchair for mobility and requires assistance for all daily activities except eating. His medical history included mitral valve replacement, permanent pacemaker implantation for complete atrioventricular block, chronic heart failure, chronic kidney disease stage G3bA3(creatinine (Cr), 1.61 mg/dL; estimated glomerular filtration rate (eGFR) 31.8 mL/min), anemia of unspecified cause, and a previous left femoral neck fracture. He took furosemide 100 mg, carvedilol 2.5 mg, atorvastatin 10 mg, ferrous citrate sodium 50 mg, clopidogrel sulfate 75 mg, rabeprazole sodium 10 mg, magnesium oxide 1500 mg, lubiprostone 24 μg, trazodone hydrochloride 25 mg, and suvorexant 15 mg as regular oral medications. Upon visiting the cardiology department, anemia was observed, with a hemoglobin (Hb) level of 6.4 g/dL (normal range for men: 13.5-17.5 g/dL), prompting referral to our department for further evaluation. The patient experienced muscle weakness preventing limb movement, yet displayed no indications of bloody stools or melena, with a notable 5kg increase in body weight from their healthy baseline. Upon examination, the patient presented with a mild level of consciousness, a body temperature of 36.5 °C, a blood pressure of 135/65 mmHg, a pacemaker-controlled heart rate of 50 bpm, and an oxygen saturation of 98% on room air. The physical examination revealed no gallop rhythm but pansystolic murmur, jugular vein distension, and indented edema on the face and lower legs. Manual muscle testing indicated a primarily decreased proximal muscle strength to 2/5, with no sensory deficits observed. Additional blood tests revealed a hematocrit (Ht) level of 19.6 % (normal range for men: 40.7-50.1 %), a mean corpuscular volume (MCV) level of 100.0 fL (normal range: 83.6-98.2 fL), a ferritin level of 380 ng/mL (normal range for men: 50-200 ng/mL), an iron level of 86 μg/dL (normal range: 40-188 μg/dL), an unsaturated iron binding capacity (UIBC) level of 123 μg/dL (normal range for men: 111-255 μg/dL), a vitamin B12 level of 573 pg/mL (normal range: 233-914 pg/mL), and a folic acid level of 3.8 ng/mL (normal range: 3.6-12.9 ng/mL). Diagnostic imaging, including a chest radiograph, revealed cardiomegaly and bilateral pleural effusion, indicative of worsening heart failure, suggestive of exacerbated heart failure (Figure 1). Initial management included the transfusion of two units of packed red blood cells and an increased oral furosemide dosage from 100 mg to 120 mg, with outpatient follow-up planned.

At a follow-up outpatient visit one week later, the hemoglobin level improved (Hb level of 7.7 g/dL); however, no improvement in symptoms was observed, and severe hypophosphatemia (serum phosphate level of 0.6 mg/dL) was detected, requiring hospitalization for further evaluation and management. Further laboratory tests confirmed severe hypophosphatemia, with serum calcium at 8.1 mg/dL, total serum calcium at 9.2 mg/dL corrected for albumin level as follows: total serum Ca = serum Ca + (4 - serum albumin; 2.9 g/dL), urinary phosphate excretion <1.0 mg/dL (below measurement limit), fibroblast growth factor 23 below the detection sensitivity, measuring less than <5.0 pg/mL (normal range: 19.9～52.9 pg/mL), intact parathyroid hormone at 20 pg/mL (below the 60-240 pg/mL target range for chronic kidney disease-mineral and bone disorder) and markedly low 25-hydroxy vitamin D levels at 8.9 ng/mL. Renal function tests showed Cr levels at 1.50 mg/dL, eGFR at 34.5 mL/min/1.73 m^2^, magnesium at 2.6 mg/dL, and brain natriuretic peptide level at 601.2 pg/ml. Echocardiography revealed a left ventricle ejection fraction (LVEF) of 34 % with reduced cardiac output, no apparent asynergy, and no new valvular disease except for previously noted moderate mitral and tricuspid regurgitation. Due to generalized edema unresponsive to diuretics and transfusion and proximal muscle weakness, symptoms of severe hypophosphatemia were strongly suspected. As a cause of the anemia, results of blood tests for creatine, iron dynamics and vitamins, responsiveness to transfusion, and lack of abnormal findings on esophagogastroduodenoscopy led to the diagnosis of renal anemia. Intravenous phosphate supplementation was initiated from 20 mmol per day, improving serum phosphorus levels to 2.4 mg/dL by day 2 of hospitalization, intravenous supplementation of 10 mmol per day was added, and oral supplementation was started from 600 mg (equal to 13.8 mmol) per day. Further to 3.6 mg/dL by day 3, supplementation was discontinued. Notably, rapid resolution of jugular vein distension and indented edema on the face and lower legs were observed following phosphorus supplementation. Laboratory and physical examination findings are shown in Tables 1, 2. Vitamin D supplementation was subsequently initiated, stabilizing serum phosphorus levels, with no recurrence of facial edema or limb weakness observed even six months after discharge. Afterward, vitamin D supplementation was initiated, leading to stabilization of the serum phosphorus levels. Even six months after discharge, there was no recurrence of facial edema, limb muscle weakness, or anemia.

**Table 1:** Characteristics of laboratory findings

Days	Day 1	Day 8	Day 10	Unit
WBC	5470	5400	5030	/μL
RBC	196	237	272	×10^4^/μL
Hb	6.4	7.7	8.7	g/dL
Ht	19.6	23.5	25.7	%
MCV	100	99.2	94.5	fL
Plt	19.7	17.5	15.8	×10^4^/μL
Alb	2.9	2.9	-	g/dL
AST	11	19	15	U/L
ALT	9	14	12	U/L
LDH	180	244	190	U/L
ALP	-	196	182	U/L
γ-GTP	-	29	29	U/L
T.Bil	0.8	0.7	-	mg/dl
Na	138	135	139	ｍEq/L
K	4.2	4.4	3.5	ｍEq/L
Cl	100	97	99	ｍEq/L
Ca	8.3	8.1	3.6	ｍEq/L
P	-	0.6	3.6	mg/dL
Mg	-	2.6	2.2	mg/dL
BUN	23.7	23.9	23.9	mg/dL
Cre	1.45	1.5	1.33	mg/dL
CRP	0.95	0.82	-	mg/dL
Ferritin	380	-	-	ng/mL
Fe	86	-	-	μg/dL
UIBC	123	-	-	μg/dL
Vitamin B12	573	-	-	pg/mL
Folic acid	3.8	-	-	ng/mL
BNP	601.2	-	-	pg/mL
FGF23	-	< 5.0	-	pg/mL
int PTH	-	20	-	pg/mL
25-OH Vit D	-	8.9	-	ng/mL

**Table 2:** Characteristics of physical examination findings

	Day 1	Day 8	Day 10
Manual Muscle Testing	2	2	4
Edema	++	++	-
Jugular venous distention	+	+	-

## Discussion

In this case, the worsening heart failure was initially suspected to be due to progressive anemia, but the patient did not respond to increased doses of diuretics and blood transfusions. The simultaneous onset of severe hypophosphatemia and proximal limb weakness and the rapid improvement in myopathy and generalized edema with phosphorus replacement led to a strong suspicion of symptoms of severe hypophosphatemia. On the other hand, a serious limitation of this case report is that we were unable to perform echocardiography before and after phosphorus replacement. Because the patient was admitted as an emergency during the night shift and treatment for symptomatic severe hypovolemia was initiated on the same day, we were unable to perform a before and after comparison. This case may have developed due to a combination of factors, including a history of chronic heart failure, as well as poor physical function, institutionalization, vitamin D deficiency, and high furosemide dose use. We also consider the possibility that the symptoms caused by severe hypophosphatemia are underestimated because phosphorus is not commonly measured. Hypothyroidism is also a differential diagnosis based on edema, weakness of proximal muscles, and anemia. In fact, TSH 10.651 μIU/mL and FT4 1.05 ng/dL met the diagnostic criteria for subclinical hypothyroidism, but since both symptoms improved with phosphorus replacement alone, symptoms due to severe hypophosphatemia were also suspected.

Symptoms such as muscle weakness and rhabdomyolysis typically present in cases of severe hypophosphatemia along with serum phosphate levels dropping below 1 mg/dL [3,8]. One possible mechanism is that decreased intracellular adenosine triphosphate concentration can impair myocardial contractility [5], and there are reports of improved cardiac function with phosphate supplementation in patients with severe hypophosphatemia [5-7]. In this case, the presence of hypophosphatemia below 1 mg/dL and the improvement of heart failure with phosphate supplementation alone, after no improvement with increased diuretics and transfusions, highly suggest hypophosphatemia as the trigger for the exacerbation of heart failure.

The primary causes of hypophosphatemia include shifting of phosphate into cells (due to insulin, sepsis, respiratory alkalosis), decreased phosphate absorption (due to vitamin D deficiency, malnutrition, alcohol use disorder, iron supplements, bisphosphonates, aluminum-containing agents), and increased phosphate excretion (due to hyperparathyroidism, vitamin D deficiency, oncogenic osteomalacia, steroids, cyclophosphamide, cisplatin, furosemide) [9,10]. Signs and symptoms of hypophosphatemia usually do not appear when the condition is solely due to a shift of phosphate into cells but become apparent in cases of chronic severe intracellular phosphate depletion [3]. Diseases causing vitamin D deficiency include hypothyroidism, liver cirrhosis, inflammatory bowel disease, post-bypass surgery, and genetic disorders, with external factors like medication (including anticonvulsants, glucocorticoids, and antiretroviral therapy), hospitalization or institutionalization, latitude, obesity, and unbalanced diet also contributing [11,12]. In this case, the likely causes of hypophosphatemia were high-dose furosemide (100 mg daily) and vitamin D deficiency due to reduced sunlight exposure under institutionalization, with the latter being more probable due to the low urinary phosphate concentration (<1.0 mg/dL, below the measurable limit), suggesting that it was primarily caused by decreased phosphate absorption due to vitamin D deficiency. Vitamin D deficiency initiates mechanisms leading to hypophosphatemia through decreased intestinal phosphate absorption and increased renal phosphate excretion caused by secondary hyperparathyroidism [13].

The lack of specific symptoms or findings for hypophosphatemia makes diagnosis and intervention difficult without measurement. Tebben et al. have suggested that serum phosphate levels should be measured in all patients with unexplained musculoskeletal symptoms [3]. People with frailty and those having decreased physical function, particularly those in institutional care, are at high risk for hypophosphatemia [2] and vitamin D deficiency [14] and an increased risk of declined physical function due to hospitalization [15]. Hypophosphatemia is likely underdiagnosed due to the lack of routine phosphorus measurements, and there may be a substantial number of undetected cases. Delays in diagnosing hypophosphatemia in frail older patients can lead to a worsening of heart failure or myopathy, resulting in delays in treatment initiation and rehabilitation and further reducing physical function. Therefore, lowering the threshold for phosphate testing is crucial as part of the diagnostic workup for unexplained worsening of congestive heart failure or proximal muscle-dominant myopathy in older patients with frailty or in institutional care.

## Conclusions

In cases of heart failure or myopathy of unknown origin, underlying hypophosphatemia can often be overlooked. Patients with underlying frailty are not only at risk of hypophosphatemia but also represent a demographic at elevated risk for complications arising during hospital stays, where delays in diagnosis or treatment can escalate the risk of further complications. Vitamin D deficiency tends to cause hypophosphatemia through decreased phosphate absorption in the intestines and increased phosphate excretion in the kidneys. In individuals at risk of vitamin D deficiency, such as those with frailty or residents of long-term care facilities, measuring serum phosphate levels should be considered to avoid delays in identifying and treating the underlying causes of heart failure or myopathy when these conditions are not immediately apparent.
